# Improving food environments and tackling obesity: A realist systematic review of the policy success of regulatory interventions targeting population nutrition

**DOI:** 10.1371/journal.pone.0182581

**Published:** 2017-08-04

**Authors:** Jana Sisnowski, Jackie M. Street, Tracy Merlin

**Affiliations:** 1 School of Public Health, University of Adelaide, Adelaide, South Australia, Australia; 2 Adelaide Health Technology Assessment (AHTA), School of Public Health, University of Adelaide, Adelaide, South Australia, Australia; Medical University of Vienna, AUSTRIA

## Abstract

**Background:**

This systematic review (PROSPERO: CRD42015025276) employs a realist approach to investigate the effect of “real-world” policies targeting different aspects of the food environment that shape individual and collective nutrition.

**Objectives:**

We were interested in assessing intermediate outcomes along the assumed causal pathway to “policy success”, in addition to the final outcome of changed consumption patterns.

**Data sources:**

We performed a search of 16 databases through October 2015, with no initial restriction by language.

**Study eligibility criteria:**

We included all publications that reported the effect of statutory provisions aimed at reducing the consumption of energy-dense foods and beverages in the general population. We allowed all methodological approaches that contained some measure of comparison, including studies of implementation progress.

**Study appraisal and synthesis methods:**

We reviewed included studies using the appraisal tools for pre-post and observational studies developed by the National Heart, Lung, and Blood Institute. Given the considerable heterogeneity in interventions assessed, study designs employed, and outcome measures reported, we opted for a narrative synthesis of results.

**Results and implications:**

Results drawn from 36 peer-reviewed articles and grey literature reports demonstrated that isolated regulatory interventions can improve intermediate outcomes, but fail to affect consumption at clinically significant levels. The included literature covered six different types of interventions, with 19 studies reporting on calorie posting on chain restaurant menus. The large majority of the identified interventions were conducted in the US. Early results from recent taxation measures were published after the review cut-off date but these suggested more favorable effects on consumption levels. Nevertheless, the evidence assessed in this review suggests that current policies are generally falling short of anticipated health impacts.

## Introduction

Regulatory measures that aim to improve population nutrition have become an increasingly popular public health strategy against obesity. As a growing number of approaches are being field tested, a new dimension of evidence has become available to inform future policy-making more realistically [1] than modeling exercises and researcher-manipulated studies in controlled settings [2, 3]. However, evaluations of early policy efforts have not been systematically and comprehensively examined. Although one recent systematic review [4] analyzed natural experiments in the areas of physical activity and nutrition, it relied on a search of PubMed only and excluded outcomes measured directly in the food environment. It reported mostly null results across the categories of interest to this study and did not identify any studies on fiscal policies or food supply measures [4].

Evaluations of policy interventions are methodologically challenging as they are necessarily observational and involve long and often indirect cause-and-effect chains that occur in parallel with a myriad of other changes in the population and environment [4–6]. Preventive interventions that target environments rather than individual behaviors present the additional difficulty that the desired impact might emerge only gradually or cumulatively in conjunction with other interventions [7]. These considerations suggest that only measuring ultimate outcomes of interest, such as changes to nutritional patterns or body weight, is not an adequate indication of policy success or failure. Instead, the impact of real-life public health interventions may be more appropriately assessed by substantiating a logical pathway connecting intervention and outcome, and by demonstrating realization of immediate program goals or the presence of more distal jurisdiction-wide trends in average weight or nutritional intake. [6–8].

In this paper, we reviewed current research evaluating real-life policy interventions addressing obesity. We used a realist review approach [9] which focused on program mechanisms to provide a more nuanced assessment of policy success or failure. Specifically, we investigated the effect of statutory provisions of a regulatory nature that aim to reduce the consumption of energy-dense foods and beverages in the general population. Outcomes included both direct (e.g. BMI) and indirect (e.g. calorie count of food choices) measures.

## Methods

**Fig 1:**
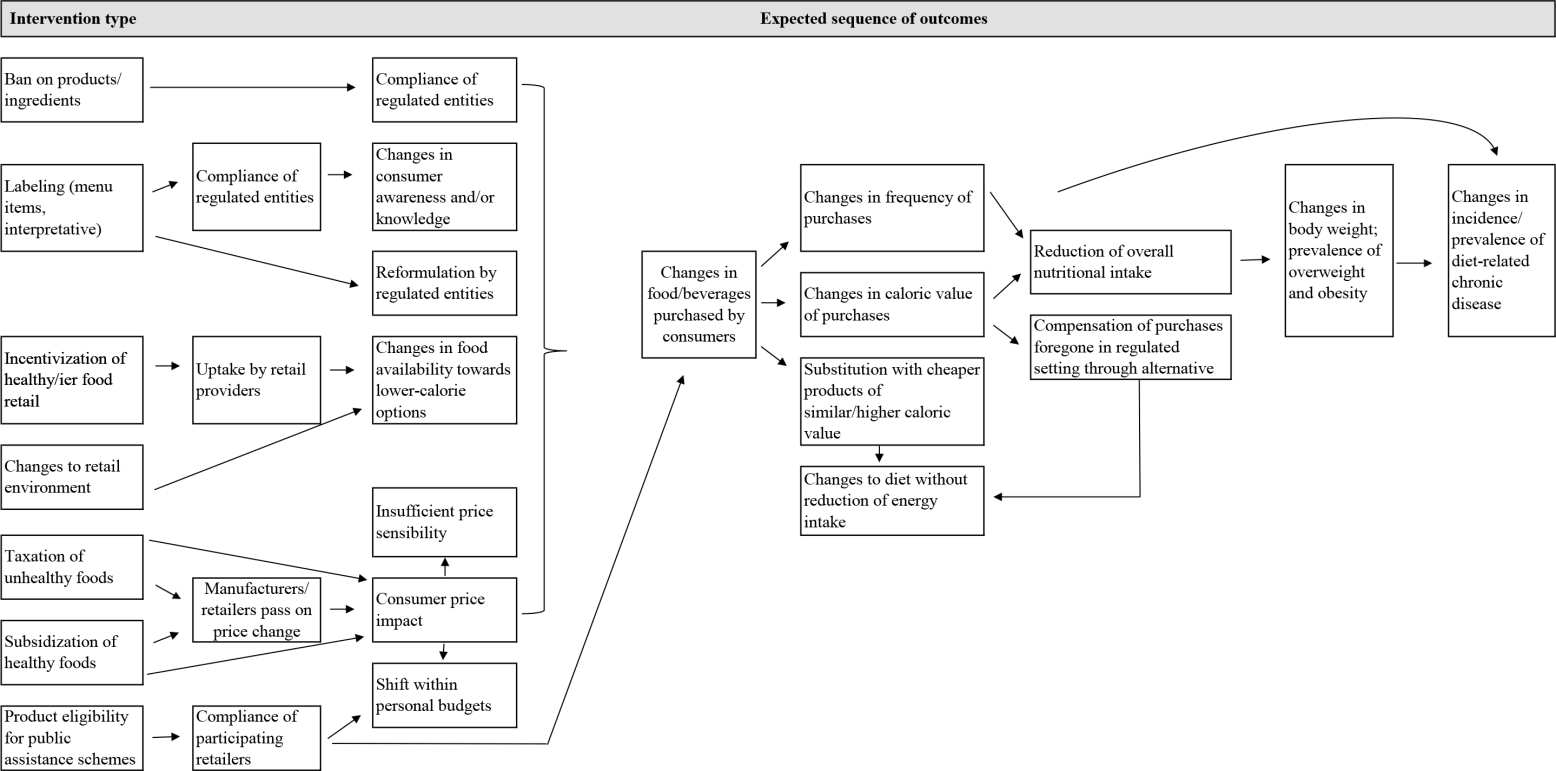
Assumed pathways from interventions to health outcomes.

Realist approaches review interventions along a continuum of indicators of successful implementation [9]. Based on a theory of the mechanisms by which an intervention is intended to bring about an intended outcome, the aim of a realist review is to provide a nuanced assessment of the extent to which interventions work and at which point of the implementation there is a failure to produce the desired end result [9]. This approach stands in contrast to the traditional review that focuses on one cumulative outcome as a maker of intervention success or failure; for instance the ability of a regulation providing information to consumers to result in changes in average BMI. Following the realist approach, the outcomes of interest for this systematic review align with the assumed program logic of interventions designed to reduce the consumption of unhealthy food and beverages and ultimately curb the prevalence of overweight and obesity. To this end, we collected data regarding (i) the effect of these interventions on average BMI or weight and on calorie intake and related proxy measures and (ii) indicators measuring parameters on the assumed causal pathway to changed consumption patterns, including measures of the degree of program implementation and non-behavioral consumer responses such as awareness and knowledge. In recognition that new policies may be evaluated on the basis of process indicators alone, we allowed all methodological approaches that included some measure of comparison, including studies of implementation progress with an assumed baseline of zero. A review protocol was developed and registered on the International Prospective Register of Systematic Reviews (PROSPERO) prior to commencement of this study (registration number CRD42015025276). As summarized in Fig 1 and in line with a realist review approach, our search and selection methods were informed by the likely program logic of interventions in the principal areas identified in the literature as possible regulatory levers [10,11].

### Data sources

We systematically searched 16 databases that span academic research as well as research undertaken by public agencies and other public or private organizations. In addition, we hand-searched the reference lists of all articles that met the study eligibility criteria detailed below. A full overview of the search strategies used in the following databases is available in the document attached to the review protocol in PROSPERO. (Also see S2 File).

### Study selection

We considered all studies published between 2004, the year WHO member states first acknowledged a role for market-related regulatory interventions for obesity prevention in the Global Strategy on Diet, Physical Activity [12] and October 31^st^, 2015 for inclusion in this review, with no initial restriction on the language of publication.

**Fig 2:**
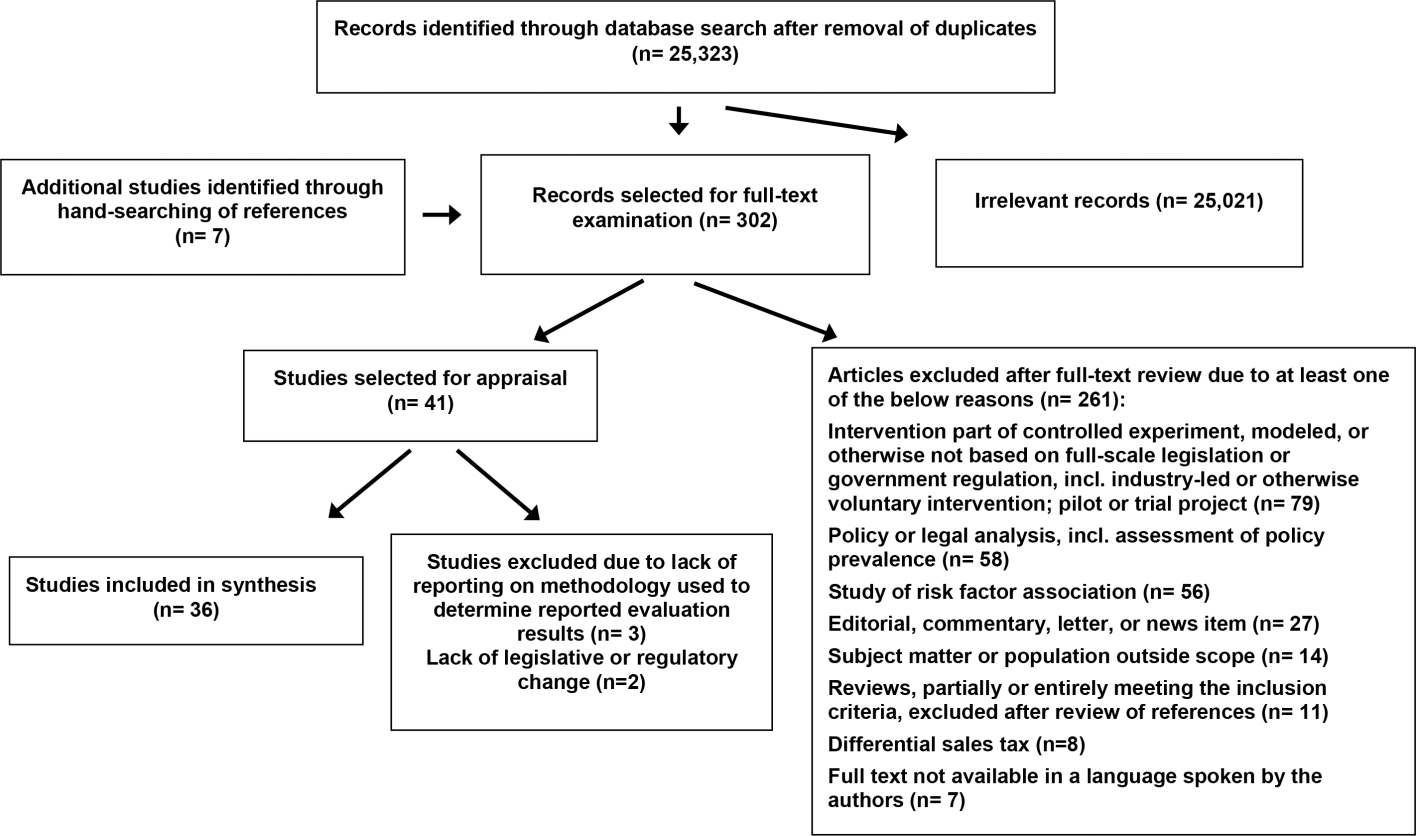
PRISMA flow diagram—Summary of search and selection process.

We included all full-scale policy interventions designed to improve population nutrition, regardless of whether the outcome(s) reported was related to the food environment or to behavioral patterns. Eligible studies (i) examined an enacted statutory intervention (ii) applied to the entire population of its jurisdiction and (iii) targeted the consumption of energy-dense foods and beverages. All interventions that are not part of a full-scale, jurisdiction-wide policy were excluded, such as pilot programs and private sector or NGO actions without a change of primary or secondary legislation. Differential sales taxes and low-level soda taxes, usually enacted solely as means to raise revenue [13], were excluded due to the lack of public health policy intent. Accordingly, eight studies reporting the effects of differential sales taxes were excluded (also see Fig 2), regardless of the presence or direction of any effect on sales levels, consumption patterns, or weight and health outcomes. In addition, we excluded all interventions aimed only at children or other defined or implicit sub-groups (e.g. school-based programs or the US Special Supplemental Nutrition Program for Women, Infants, and Children), but retained those that provide a social safety net open to anybody in demonstrated need (e.g. the US Supplemental Nutrition Assistance Program (SNAP)/ food stamp program).

After removal of duplicates, we screened 25,323 items for relevance according to the inclusion criteria. The first reviewer (JS) initially assessed each title and, where available, abstract. A subset of 10% of the initial search results was again reviewed for eligibility according to the inclusion criteria by a second reviewer (JMS). Where study eligibility was disputed, the co-authors reached a consensus decision. The first reviewer then retrieved and assessed the full text of 302 articles that had been determined to possibly meet the inclusion criteria in the first round of screening. In addition to studies reporting on the evaluation of one specific intervention matching the inclusion criteria, we also retained eleven systematic reviews whose inclusion criteria overlapped at least partially with ours. We reviewed the reference lists of these reviews for additional eligible studies before excluding the reviews themselves from further analysis. Together with the hand-searching of the reference lists of all included studies, this process yielded an additional seven eligible articles. The same two reviewers independently assessed the 48 selected studies for methodological quality prior to inclusion in the review using the appraisal tools for Quality Assessment of Before-After (Pre-Post) Studies With No Control Group [14] and for Quality Assessment of Observational Cohort and Cross-Sectional Studies [15] developed by the National Heart, Lung, and Blood Institute. At this stage, we excluded a further three studies which reported evaluation outcomes, but did not detail or reference the underlying methodology. An additional two studies assessing health department-led initiatives aimed at improving the availability of healthier food in corner stores in New York City and Philadelphia were excluded due to these interventions being programmatic in nature without a change in legislation or regulation. By contrast, studies reporting on the effect of local subsidies for healthy purchases made under the US Supplemental Nutrition Assistance Program (SNAP) are considered within scope of the inclusion criteria given that incentive programs targeting SNAP recipients are subject to the laws and US Department of Agriculture (USDA) regulations governing the SNAP scheme and, at the time the interventions under evaluation were implemented, required jurisdictions to obtain formal approval from the USDA Food and Nutrition Service. The flow diagram in Fig 2 below summarizes the database search and study selection process. Also see Prisma Checklist, S3 File)

### Data extraction

We grouped studies according to type of intervention reported and extracted the following details for each reference: setting, study design, time post-implementation, main population and, where applicable, sub-populations, results for the primary outcome, and, where applicable, results for any secondary outcomes reported.

## Results

The 36 studies [16–53] span six different types of interventions: a majority (n = 19) report on calorie posting on chain restaurant menus, followed by changes to food infrastructure (n = 5), subsidies for healthy food purchases (n = 5), taxation of unhealthy foods and beverages (n = 5), government food standards (n = 1), and nutrition labeling of products (n = 1). Approximately 80% of included studies (n = 30) assessed interventions implemented in the United States. Evaluation strategies varied and resulted in different endpoints, often with multiple strategies used in one report to quantify the success of program implementation and the effects on behavior. Methodological quality against the Quality Assessment of Before-After (Pre-Post) Studies With No Control Group [14] and the Quality Assessment of Observational Cohort and Cross-Sectional Studies tools varied between studies. The majority of studies were judged to be of medium quality. Recurring limitations were related to limited sampling frames and overly descriptive approaches. Methodological limitations are considered as part of the narrative synthesis below and further discussed in the discussion and conclusion section. The results table in the annex (S1 File) provides a detailed overview of the included studies, including quality appraisal. In the following, we summarize the results in a narrative meta-synthesis.

### Menu labeling

Calorie posting on menus at chain restaurants has been the most comprehensively examined intervention of the approaches identified in this study. Our review identified 19 individual studies with predominantly pre-post designs or repeat cross-sectional surveys with a control group, with sample sizes ranging from a few hundred participants [16,23,29,32] to over 100 million transactions [17]. In the following, we synthesize the study results by the type of outcome examined, starting with the outcomes most distal from the intervention in accordance with the program logic outlined in Fig 1 in the methods section (Fig 1). Note that several studies reported more than one outcome measure.

#### (1) Changes in calorie value of purchases

Studies measuring average calorie intake, based on verified purchases or self-reported consumption and not restricted to one restaurant chain, suggest that menu labelling using calorie per item does not impact on consumer purchasing behavior [21,23–27,29,31–33]. However, two studies reported a differential post-implementation drop in average calories ordered. Both took place at only one regulated chain. The first used two outlets of a non-identified fast food chain in Philadelphia and control locations in neighboring states, resulting in a total sample of 648 verified purchases. This study reported a 9% drop, equivalent to 151 food calories less purchased on average compared to non-regulated jurisdictions [16]. The second study was limited to the Starbucks chain in New York City, with Boston and Baltimore as control locations [17]. It was one of two evaluations [17,26] in which a chain agreed to share its sales data. Starbucks sales data showed a drop of 6% to an average order of 232 calories post-implementation [17]. However, the caloric value of average purchases at Starbucks were much lower than at other regulated chains both pre- and post-implementation. For instance, in the other single chain study, customers at regulated outlets purchased an average of 1,556 calories [16] and the average entrée in King County contained 777 calories at 18 months post-labelling before adding any side orders [18]. This suggests that the Starbucks study may not be representative of the regulation’s impact in the broader fast food sector. Interestingly, the Starbucks study also observed that the company’s aggregate sales revenue remained stable post-implementation and even increased by 3% at stores located near a Dunkin Donuts [17]. Assuming that the increase in sales near rival outlets indeed represents a shift of customers rather than new customers, it seems that the chain attracts more health-conscious consumers away from equally regulated competitors. Taken together, these two observations call into question the external validity of the Starbucks study.

#### (2) Changes in frequency of visits to fast food restaurants

The idea that an effect might occur outside the restaurant setting and therefore be undetectable in cross-sectional studies was investigated four months after Philadelphia’s introduction of menu labeling [25]. Program logic indicates that potential fast food consumers might respond to the new labeling by reducing the number of restaurant visits without changing the amount of calories at each visit. However, the study found no reduction in the number of fast food restaurant visits by either consumers intercepted at a fast food restaurant or by those questioned in a random-digit dialing phone survey [25]. While there was no statistically significant association in either direction, trends across several sub-groups suggest that if there was an effect, it would more likely trend towards an increased number of average visits post implementation [25].

#### (3) Changes in consumer knowledge

Intermediate outcomes on the logical pathway to consumption were frequently measured as the sole endpoint [19,20,22,31] or as a secondary outcome [16,21,23–25,27,29,32,33]. Changes in measures such as self-reported noticing of calorie labels and self-reported usage in ordering varied by location and study: for instance, the average share of consumers reporting having noticed calorie labeling at the end of the respective post-implementation observation period ranged from 38% to 76% in Philadelphia [25,16], from 58% to 59% in Washington State [19,27], and from 54% to 64% in NYC [24,20]. Similarly, 57% of adolescents in NYC [23] and 87% of parents ordering for their children in Washington State reported noticing calorie labels after their introduction [32].

Across all studies, the share of customers who reported using the calorie information in purchasing decisions was far below the share noticing it. Among those making use of the labeling, uptake tended to vary by sub-population, but showed few consistent trends across studies. For instance, in a Washington State study women, high income earners, and whites had greater odds of using menu labeling [19] and in a second Washington State study usage differences were found between women and men, but not between races or ethnicities [27]. In NYC, men were more likely than women to report using the information [20], with the opposite finding reported in another study also conducted there [21].

In addition to self-reported usage of information, a more objective measure of the successful translation of information provision to nutrition knowledge was reported by one study: in New York City, the proportion of respondents correctly estimating the caloric value of their purchase rose from 15% to 24%, while declining from baseline in the control city of Newark, resulting in a statistically significant differential change during the post-implementation period [22]. However, no statistically significant differential change in correct estimates of recommended daily calorie intake was reported post-implementation [22], suggesting that customers still lacked a reference against which to judge the calorie content of their meals. In Philadelphia, differential changes in the accuracy of estimates of calories purchased were statistically significant only in customers with at least some college education and in those ordering small meals, perhaps a sign of greater health consciousness in those customers [31].

#### (4) Reformulation by regulated chain restaurants

It appears that at least in Washington State, where King County implemented a new menu labeling regulation, chain restaurants responded to the change through modest reformulation of their menus, thus bypassing consumer decision-making on the pathway to reduced calorie intake [18]. On average, entrées contained 41 calories less at 18 months after enactment of the new rule compared to at six months, a 5% drop to 777 calories per entrée [18]. A comparison of menus between chains operating in regulated jurisdictions and chains operating only in non-regulated jurisdictions showed that the availability of healthier food options increased by 8% at regulated chains, but remained constant at control chains [28]. No difference in average caloric content was found between regulated and control menus [28]. Another King County study looked at the wider restaurant environment post-implementation and found few qualitative changes to the food environment other than compliance with the use of regulation compared to a control jurisdiction [30]. Overall, these studies imply broad compliance with the regulations, but show only small spillover effects into other aspects of the restaurant food environment.

#### (5) Policy diffusion through convergence of practice

One study from Australia provides insights into the possible effect of policy innovation across jurisdictional borders. Conducted one year before and 11 months after New South Wales (NSW) became the first state to introduce mandatory menu labeling, the study reports on nationwide trends for the five fast food chains with the largest numbers of outlets in Australia [34]. The study design neglected to compare NSW with the non-regulated states and is therefore of little use to assess the implementation of the regulation in NSW. However, the study reported that the average total nutrition information available in stores rose significantly across the nation while the number of outlets with no nutrition information available dropped by 31% to just two stores in the sample. This finding attests to the power of policy diffusion through convergence of practice in nationally and internationally operating food businesses.

### Improvement of food infrastructure

Studies reporting on the success of changes to the food infrastructure are limited to the two US jurisdictions of New York City and South Los Angeles in Los Angeles County.

New York City’s Green Carts program made available up to 1,000 permits for mobile vendors of fresh produce in specified disadvantaged neighborhoods. The program did not result in any statistically significant increase in reported fruit and vegetable consumption [36]. Vendors tended to cluster along public transport, commercial, and other hubs within their designated zones and thereby largely bypassed the most disadvantaged neighborhoods [37,38]. In addition, not all 1,000 permits were taken up: two evaluations found approximately 50% of permits active on paper [35,36], but when attempting to locate all vendors, only 166 carts could be located [35].

Meanwhile, South Los Angeles’ ban on new free-standing fast food chain outlets also showed limited effectiveness in improving the food environment. Four and a half years after implementation, only 10% of food outlets operating at the time of the study had opened under the new rule [39]. This indicates limited reach of a law applying only to new businesses in a fairly stable food environment. Not surprisingly, after controlling for individual and collective characteristics, the study found no statistically significant differences in diet and BMI changes in comparison to control jurisdictions [39].

### Subsidies for healthy foods

These studies examined the use of subsidies centered on the US Supplemental Nutrition Assistance Program (SNAP), formerly known as food stamps, and local efforts to incentivize their use for the purchase of healthy foods. At the aforementioned Green Carts in NYC, the use of SNAP benefits was associated with an average of $3.86 more spent compared to cash payment [41]. The Health Bucks program in NYC and the Philly Food Bucks program in Philadelphia both offered $2 vouchers per $5 in SNAP benefits spent at farmers’ markets. Both programs resulted in increased SNAP sales at farmers’ markets (40,44). In addition, vendors in NYC reported a high degree of satisfaction with the impact of the program on their business [43]. Although voucher users in Philadelphia were 2.6 times more likely to report increased fruit and vegetable consumption since becoming market customers than non-voucher users [44], health survey data in NYC showed no differential increase in self-reported fruit and vegetable consumption after introduction of the program compared to control neighborhoods [42].

### Taxation of unhealthy foods and beverages

Taxation of unhealthy foods and beverages expressly for public health purposes represented the only category not dominated by US evidence. All five studies in this category investigated European approaches. The French beverage tax of 7.16 euros per hectoliter (0.076 euros per liter) was passed through fully to retail prices for soda and partially for other taxed beverage categories at six months post-implementation [45], thereby validating the first step on the logical pathway to reduced consumption of sweetened beverages.

Three studies [47–49] quantified the effects of the now abolished Danish tax on saturated fat content and concluded that there was an effect on consumption levels as measured by proxy sales and purchasing data. A study based on a panel of 2,000 households found that purchases of butter, butter blends, margarine and oils decreased by 10%-15% in the first nine months post-implementation [48]. However, this was at least partially attributed to hoarding prior to the entry into force of the new tax [48]. A study of sales data collected from different retail chains owned by Coop Denmark showed different price developments for the three product groups (minced beef, cream, and sour cream) up to one year post-implementation [49]. Prices of minced beef and cream were higher post-implementation, but no consistent pattern was observed for sour cream prices. In addition, price changes were stronger for the medium-fat and weakest for the low-fat varieties of minced beef and cream [49]. Matching the price changes, sales changes suggested there was a decrease of 4–6% in the intake of saturated fat from minced beef and cream, while no significant effect was found for sour cream [49]. Another study examined sales data for twelve taxed foodstuffs over the entire 15 months of the tax’s existence [47]. It reported a total decrease in sales across product categories by 0.9%, but an increase by 1.3% pre-implementation and post-abolition of the tax. One modelling approach estimated that sales changes could translate into a population-wide increase in the incidence of ischemic heart disease by 0.2% due to a decrease of both harmful saturated fat and beneficial unsaturated fat intake [47]. Although this is only one of two possible estimates (the other one forecasting a total decrease of 0.3%) and a very small effect size, this result illustrates that targeting specific nutrients in a wide range of foodstuffs may entail unintended changes in consumption patterns that mitigate or negate the intended effects.

In Hungary, a broad-based junk food tax was estimated to have reduced purchases of processed foods, which were mostly taxed, by 3.4%, while purchased quantities of unprocessed foods increased by a statistically insignificant 1.1% at 16 months post-implementation [46].

### Procurement standards for public institutions

The only full-scale evaluation of a jurisdiction setting standards for the nutritional quality of items available to its employees and the general public is that of the Healthy Beverage Executive Order enacted by the city of Boston [50]. Two years post-implementation, unhealthy beverage availability and average caloric content per beverage declined considerably compared to the pre-implementation period and compared to control sites. Control sites were owned by the city and the state of Massachusetts and not covered by the order. Compared to the pre-implementation period, access to red-coded, unhealthy beverages decreased by 27.8% (P<0.001) overall; red beverage access in vending machines decreased by 28.9% (P<0.001) and in cafes/cafeterias by 20.4% (P = 0.02). In addition, average calories per beverage sold within access points decreased by 48.6 kcal from 88.1 kcal to 39.5 kcal post-implementation [50]. However, positive trends of a lesser magnitude were also observed at the comparison sites, particularly the ones owned by the city rather than the state, indicating that a larger trend or a signaling effect beyond the direct intervention may have contributed to the changes.

### Nutrition labeling of products

The only study identified that assessed nutrition labeling on products originated in New South Wales, an Australian state [51]. Reporting that only 7% of 350 product samples matched the exact nutritional information given on the label in a laboratory test [51], this study is narrowly focused on compliance. However, as interpretive labeling approaches are increasingly considered, it does raise the question to what extent nutrition labeling can be enforced beyond adherence to design and presentation rules and what constitutes an acceptable margin of error for consumer information.

## Discussion and conclusion

These findings indicate that isolated regulatory interventions frequently result in improvements of the most proximal outcomes, measured in the food environment and situated at the very beginning of the logic model. However, the interventions assessed here fail to achieve an effect on consumption that could plausibly be considered as clinically significant, i.e. as having an effect on individuals’ nutritional intake to the extent that it would reduce the incidence of overweight, obesity, and related chronic diseases. This is a differentiation between different levels of policy success and failure that have not been highlighted previously [4].

When compared to just a few examples of effect estimates put forward during policy development and decision-making processes, it is clear that current interventions are falling short of the public health impact hoped for by policy-makers and predicted by many researchers. For example, in New York City, the Department of Health estimated that the new calorie posting rule would lead to “at least 150,000 fewer New Yorkers [becoming] obese, [and] at least 30,000 fewer cases of diabetes” [52] over five years. However, with the exception of the study focused on Starbucks, the New York City-based studies profiled here failed to find statistically and clinically significant calorie reductions [21–24,33]. Moreover, a recent look at the sustained impact of the intervention, published just after the cutoff date for this review, concluded that even minimal improvements in consumer awareness appear to have diminished over time [53]. Meanwhile, the Danish forecast that a fat tax would eventually add 5.5 days to the average Dane’s lifespan [54] will remain unrefuted given the quick abolition of the measure, but appears tenuous given the early evaluation results. Similarly, one of the evaluations of New York City’s Green Cart program reported that the city originally estimated that the generated increased intake of fruits and vegetables would measurably improve the health status of 75,000 individuals and avert loss of at least 50 lives a year [35]. However, program administrators concluded that the direct impact of the intervention on morbidity and mortality would be too difficult to quantify and the program evaluators observed that direct health-enhancing arguments for Green Carts subsequently faded [35].

This is not to say that these interventions may not deliver cumulative behavioral and health effects in the long-term, especially where they act in parallel with complementary interventions and change social and political perceptions of nutrition. In this context, it is notable that recent studies at national level and in hotspots of obesity prevention activities such as New York City have found both a shift in the attitudes of consumers towards sugary drinks and an actual reduction in average soft drink consumption [55–57].

Some of the interventions discussed above may also be inconsequential and not have a meaningful impact on consumption: a recent review of regulation targeting sugar-sweetened beverages argued that policies are squandering the potential for more pronounced behavioral impact because of their restrained design, possibly to appease industry and political opponents [58]. Indeed, very few of the above interventions match the designs identified in the literature as the more effective public health approaches, be it displays of physical activity equivalents instead of plain calorie counts [59] or excise taxes amounting to price increases of at least 15–25%, equivalent to the long advocated penny per ounce tax [60–62]. In 2015, Berkeley, California, passed a tax on sugar-sweetened beverages that matched the magnitude suggested by public health experts and in 2014, Mexico implemented a tax of one peso per liter which, if passed on to consumers, comes close to the recommended level with a 10% increase in price. Two early evaluations, published just after the cut-off date for this review, reported that in both locations, the taxes were generally passed on, with higher price increases relative to taxation levels reported for Mexico compared to Berkeley [63,64]. The study in Berkeley employed comparison cities for control of pre-post trends and reported pass-through rates of 69% for soda and 47% for all taxed products [63]. Neither jurisdiction reported deleterious effects on the prices of non-taxed beverages such as bottled water, with the exception of slight price increases for diet soda in Berkeley [63,64]. A third evaluation of the Mexican tax published in 2017 indicated that purchases of taxed beverages fell by 7.6% in the two years after implementation of the tax compared with a 2.1% drop in untaxed beverage purchases over the same period [65]. These two fiscal interventions warrant close attention from the experts and policy-makers as they represent rare examples of current policy recommendations being put into practice. Most recently, additional jurisdictions have approved soda taxes, among these decisions by the legislative bodies of Philadelphia City and Cook Country, which includes Chicago, as well as popular votes in San Francisco, Oakland, and Albany in California and in Boulder in Colorado [66]. Both the British and Irish governments have announced plans for the introduction of soda taxes in 2018 [66].

Some limitations must be taken into account when interpreting the results of this systematic review. Firstly, despite an expansive search of a variety of databases and broad inclusion criteria, it is likely that some evaluations of real-life interventions are not available through academic and gray literature repositories. It is reasonable to suppose that in many instances, especially in lower level jurisdictions and in middle and low-income countries, no formal evaluation of relevant policies would have been undertaken and/or reported. As a result, only a small number of studies from outside the OECD were identified, with several articles describing interventions in Ghana [67] and the Pacific island region [68,69] excluded at the appraisal stage due to the unavailability of detailed evaluation processes and results. Similarly, it is possible that unsuccessful policy interventions remain underreported and those that are published put a greater emphasis on intermediate program outcomes that show greater progress than the actual policy end goal. These limitations underline the need to make methodically sound evaluations a routine component of policy implementation and highlight the usefulness of some form of centralized repository for comprehensive evaluation reporting that is accessible globally.

Secondly, our study purposely used appraisal tools tailored to observational study designs that are amenable to evaluating real-life policies. As outlined in the introduction, real-life policy experiments do not always fully align with the methodological expectations of evidence-based health sciences, particularly when compared to targeted prevention delivered in health care settings. This issue was raised in relation to the study by Dumanovsky and colleagues who employed a simple pre-post study design to quantify the effect of menu labeling in New York City [21]. Criticized for the chosen study design [70], the authors responded that the methodology needs to match both the reality of a policy in progress and the limited resources of a public agency carrying out its own evaluation while stymied by the refusal of industry to share its sales data [71]. Despite some reservations about these study designs expressed in the literature, studies using conventional cross-sectional designs or simple pre-post designs posed little difficulty for appraisal and data extraction. Conversely, studies that evaluated implementation processes were problematic to appraise and summarize. Aspects of design, common in the evaluation of food infrastructure interventions such as the SNAP subsidies in Philadelphia and NYC and NYC’s Green Cart program, complicated the assessment of studies examining food infrastructure improvements and food subsidization programs. These aspects included the use of descriptive approaches and a mix of different study designs of varying quality within single reports. The difficulties that we encountered suggest that scholarly assessment of study quality and the reality of policy-making in perennially resource-constrained health departments occasionally collide. As a result, even more differentiated appraisal tools need to be used for evaluation in recognition that studies with descriptive approaches can be useful for charting implementation progress by ensuring that program logic is in place.

To conclude, our review underlines that the immediate expectations associated with the examined types of regulatory interventions need tempering. At this point in time, the policy examples discussed above primarily deliver proof of feasibility: the fact that they survived the policy-making process and have been mostly successful in reaching immediate program goals should enhance the political palatability of such approaches even if, at the time of examination, there has been little demonstrated impact on risk factors and health outcomes. Policy-makers should therefore not dismiss such recent policy experiments as failures, but pursue the example of these jurisdictions as necessary building blocks for more stringent and comprehensive nutrition policy and obesity prevention regimes.

## Supporting information

S1 File Detailed overview of included studies. (DOCX)

S2 File Overview of database search strategies. (DOCX)

S3 File Prisma checklist. (DOC)
