# Supplementary material for: Improving food environments and tackling obesity: A realist systematic review of the policy success of regulatory interventions targeting population nutrition
Source: PLoS One. 2017 Aug 4;12(8):e0182581. doi: 10.1371/journal.pone.0182581 (PMC5544242; doi:10.1371/journal.pone.0182581)
Supplement: S1 File — (DOCX) [file pone.0182581.s001.docx]

**S1 File: Improving food environments and tackling obesity: a realist systematic review of the policy success of regulatory interventions targeting population nutrition - Overview of database search strategies**

1. **PubMed (n=8,995)**

| January 1, 2004 – October 31, 2015; humans; string:(A AND (B OR C OR D OR E)) NOT E | |
| --- | --- |
| A  Policy/intervention | (Policy[tiab] OR policies[tiab] OR regulation[tiab] OR regulations[tiab] OR regulatory[tiab] OR Government Regulation[MH] OR prevention & control[MH] OR Risk Reduction Behavior[MH:noexp] OR Policy[MH:noexp] OR Public Policy[MH:noexp] OR Health Policy[MH:noexp] OR Nutrition policy[MH] OR intervention[tiab] OR interventions[tiab] OR law[tiab] OR laws[tiab] OR legislation[tiab] OR legislative[tiab] OR legal[tiab] OR health promotion[tiab]) |
| B  Obesity | (obesity[tiab] OR obese[tiab] OR adiposity[tiab] OR adipose[tiab] OR overweight[tiab] OR weight[tiab] OR bodyweight[tiab] OR Overnutrition[MeSH Terms] OR Body Weights and Measures[MeSH Terms] OR Body Weight[MeSH Terms] OR BMI[tiab] OR body mass index[tiab] OR Overweight[MeSH Terms] |
| C  Nutrition | nutrition[tiab] OR nutritional[tiab]OR diet[tiab]OR dietary [tiab] OR diets [tiab] OR food[tiab] OR foods[tiab] OR calorie [tiab] OR calories [tiab] OR calorie-dense[tiab] OR calorie dense[tiab] OR caloric [tiab]) OR energy [tiab] OR energy-dense[tiab] OR energy dense[tiab] OR intake [tiab] Or Energy Intake[MH] OR consumption [tiab] OR eating[tiab] OR Food Habits[MH] OR Food Preferences[MH] OR Nutritional Status[MH] |
| D  Foods/food components | vegetable[tiab] OR vegetables[tiab]OR fruit[tiab] OR fruits[tiab] OR sugar[tiab] OR sugars[tiab] OR sugary[tiab] OR fat[tiab]) OR fats[tiab] OR fatty [tiab] OR fast food[tiab] OR junk food[tiab] OR drink[tiab] OR drinks[tiab] OR beverage[tiab] OR beverages[tiab] OR Food and Beverages[MH] |
| E  Infrastructure and settings | menu[tiab] OR menus[tiab] OR label[tiab] OR labels[tiab] or labeling[tiab] OR labelling[tiab] OR restaurant[tiab] OR restaurants[tiab] OR grocery[tiab] OR groceries[tiab] OR store[tiab] OR stores[tiab] OR bodega*[tiab] OR supermarket*[tiab] OR market[tiab] OR markets[tiab] OR greenmarket[tiab] OR greenmarkets[tiab] OR tax[tiab] OR taxes[tiab] OR taxation[tiab] OR Taxes[MH:noexp] OR subsidy[tiab] OR subsidies[tiab] OR subsidization[tiab] OR subsidisation[tiab] OR zoning[tiab] OR density[tiab] OR procurement[tiab] |
| F  Publication Type | Clinical Trial [Publication Type] OR Controlled Clinical Trial [Publication Type] OR Randomized Controlled Trial [Publication Type] OR randomized[tiab] OR randomised[tiab] OR Comment [Publication Type] OR Editorial [Publication Type] OR Letter [Publication Type] OR News [Publication Type] OR Newspaper Article [Publication Type] |

1. **Embase (n= 6,254)**

| Search string: (#1 AND (#2 OR #3 OR 4 OR #5) AND ([article]/lim OR [article in press]/lim OR [review]/lim) AND [humans]/lim AND [embase]/lim AND [2004-2015]/py) NOT #6 | |
| --- | --- |
| #1  Policy/intervention | 'policy'/syn OR policy:ab,ti OR policies:ab,ti OR 'public policy'/mj OR 'public policy':ab,ti OR 'health policy'/mj OR 'health policy':ab,ti OR 'nutrition policy'/mj OR 'nutrition policy':ab,ti OR 'law'/mj OR law:ab,ti OR laws:ab,ti OR 'legislation'/mj OR legislation:ab,ti OR legislative:ab,ti OR legal:ab,ti |
| #2  Obesity | 'obesity'/syn OR obesity:ab,ti OR obese:ab,ti OR overweight:ab,ti OR bodyweight:ab,ti OR 'body weight'/syn OR 'body weight':ab,ti OR bmi:ab,ti OR 'body mass index'/mj OR 'body mass index':ab,ti |
| #3  Nutrition | 'nutrition'/syn OR nutrition:ab,ti OR nutritional:ab,ti OR diet:ab,ti OR dietary:ab,ti OR diet:ab,ti OR food:ab,ti OR foods:ab,ti OR calories:ab,ti OR 'calorie dense':ab,ti OR 'calorie'/mj OR calorie:ab,ti OR caloric:ab,ti OR 'energy dense':ab,ti OR intake:ab,ti OR energy:ab,ti OR consumption:ab,ti OR ‘feeding behavior’/exp OR eating:ab,ti OR ‘dietary intake’/exp |
| #4  Foods/food components | 'vegetable'/mj OR vegetable:ab,ti OR 'vegetables'/mj OR vegetables:ab,ti OR 'fruit'/mj OR fruit:ab,ti OR fruits:ab,ti OR 'sugar'/mj OR sugar:ab,ti OR sugary:ab,ti OR 'sugar sweetened':ab,ti OR 'fat'/mj OR fat:ab,ti OR 'fast food'/mj OR 'fast food':ab,ti OR 'junk food':ab,ti OR drink:ab,ti OR drinks:ab,ti OR 'beverage'/mj OR beverage:ab,ti OR 'beverages'/mj OR beverages:ab,ti |
| #5  Infrastructure and settings | menu:ab,ti OR menus:ab,ti OR label:ab,ti OR labels:ab,ti OR labelling:ab,ti OR labeling:ab,ti OR restaurant:ab,ti OR 'restaurants'/mj OR restaurants:ab,ti OR grocery:ab,ti OR groceries:ab,ti OR store:ab,ti OR stores:ab,ti OR bodega*:ab,ti OR supermarket*:ab,ti OR 'market':ab,ti OR markets:ab,ti OR greenmarket:ab,ti OR greenmarkets:ab,ti OR 'tax':ab,ti OR 'tax'/mj OR taxation:ab,ti OR 'taxes':ab,ti OR 'taxes'/mj OR subsidy:ab,ti OR subsidies:ab,ti OR subsidisation:ab,ti OR subsidization:ab,ti OR zoning:ab,ti OR 'density':ab,ti OR procurement:ab,ti OR 'food assistance'/mj OR 'food packaging'/syn OR 'food availability'/syn |
| #6  Exclusions | 'clinical trial':ab,ti OR rct:ab,ti OR 'gene' OR 'gene'/de OR gene OR 'genes' OR 'genes'/de OR genes OR gene:ab,ti OR genetic:ab,ti OR 'cell' OR 'cell'/de OR cell OR 'cells' OR 'cells'/de OR cells OR 'absorption' OR 'absorption'/de OR absorption OR 'mutation' OR 'mutation'/de OR mutation OR hormone:ab,ti OR hormonal:ab,ti OR 'physical activity':ab,ti OR 'physical activity'/exp OR 'physical activity'/de OR 'physical activity' OR 'exercise' OR 'exercise'/de OR exercise OR chemistry:ab,ti OR 'surgery'/exp OR 'surgery'/de OR 'surgery' OR organ* OR 'alcohol' OR 'alcohol'/de OR alcohol OR 'drinking' OR 'drinking'/de OR drinking OR 'transplantation' OR 'transplantation'/de OR transplantation OR medicine:ab,ti OR medicines:ab,ti OR 'drug' OR 'drug'/de OR drug OR 'drugs' OR 'drugs'/de OR drugs OR 'addiction' OR 'addiction'/de OR addiction OR hospital:ab,ti OR hospitals:ab,ti OR 'malnutrition' OR 'malnutrition'/de OR malnutrition OR 'undernutrition' OR 'undernutrition'/de OR undernutrition OR 'cancer' OR 'cancer'/de OR cancer |

1. **CINAHL (n=8,160)**

| ( S1 AND ( (S2 OR S3 OR S4 OR S5) ) ) NOT S6  Limiters - Published Date: 20040101-20151031; Publication Type: Doctoral Dissertation, Journal Article, Meta Analysis, Meta Synthesis, Research, Review, Systematic Review; Search modes - Boolean/Phrase; Source types- Academic Journals, Dissertations | |
| --- | --- |
| S1  Policy/intervention | (MM "Health Policy+") OR (MM "Policy Studies+") OR (MM "Policy Making") OR (MM "Health Policy Studies") OR (MM "Nutrition Policy+") OR (MM "Public Policy+") OR "policy" OR "policies" OR (MM "Legislation") OR "legislation" OR "legislative" OR "law" OR "laws" OR "legal" OR (MH "Public Health Nutrition") |
| S2  Obesity | (MH "Obesity/LJ/EV/PC/RF") OR "obesity" OR "obese" OR "overweight" OR (MH "Body Weight/EC/EV/LJ/ST/TD") OR (MH "Weight Control/EC/EV/LJ/ST") OR "bodyweight" OR "weight" |
| S3  Nutrition | (MH "Nutrition") OR "nutrition" OR "nutritional" OR "diet" OR "dietary" OR "food" OR "foods" OR "calorie" OR "calories" OR "caloric" OR "calorie-dense OR "energy-dense" OR "intake" OR "consumption" OR "eating" OR (MH "Food Preferences/EV/PC/LJ") OR (MH "Food Intake") OR (MH "Energy Intake") OR (MH "Energy Density") OR (MH "Portion Size") OR (MH "Food and Beverages") |
| S4  Foods/food components | (MH "Fruit/EC/LJ/ST/SD/UT") OR "fruit" OR "fruits" OR (MH "Vegetables/EC/LJ/ST/UT") "vegetable" OR "vegetables" OR "sugar" OR "sugary" OR "sugar-sweetened" OR "fat" OR (MH "Fast Foods/EC/LJ/ST/SD/UT") OR "fast food" OR junk food" (MH "Fruit/EC/LJ/SD/ST") OR "junk food" OR "drink" OR "drinks" OR "soda" OR "beverage" OR "beverages" |
| S5  Infrastructure and settings | "menu" OR "menus" OR (MH "Food Labeling/EC/EV/LJ/ST/SN/UT") OR "label" OR "labels" OR "labelling" OR "labeling" OR "restaurant" OR "restaurants" OR "grocery" OR "groceries" OR "store" OR "stores" OR "bodega" OR "supermarket" OR "market" OR "markets" OR "greenmarkets" OR "greenmarkets" OR (MH "Taxes") OR "tax" OR "taxation" OR "taxes" OR (MH "Food Assistance/AM/EC/EV/LJ/PC/ST/TD") OR "subsidy" OR "subsidies" OR "subsidisation" OR "subsidization" OR "zoning" OR "density" OR "procurement" |
| S6  Exclusions | (MH "Clinical Trials+") OR (MH "Randomized Controlled Trials") OR "clinical trial" OR "RCT" OR (MH "Physical Activity") OR (MH "Physical Fitness+") OR (MH "Exercise+") OR (MH "Sports+") OR "physical activity" OR "exercise" OR (MH "Genes+") OR (MH "Genetic Research+") OR "gene" OR "genes" OR "genetic" OR (MH "Cells+") OR "cell" OR "cells" OR "absorption" OR “mutation” OR (MH "Hormones+") OR "hormone" OR "hormonal" OR "chemistry" OR "surgery" OR "organ" OR "organs" OR "organic" OR "alcohol" OR (MH "Alcohol-Related Disorders+") OR (MH "Alcohol Drinking+") OR (MH "Surgery, Operative+") OR "transplantation" OR "medicine" OR "medicines" OR (MH "Drugs+") OR (MH "Therapeutics+") OR (MH "Diagnosis+") OR "drug" OR "drugs" OR "addiction" OR (MH "Health Facilities+") OR "school" OR "schools" OR "hospital" OR "hospitals" OR "malnutrition" OR "undernutrition" OR "cancer" |

1. **PsycINFO (n=2,074)**

| (#1 and (#2 or #3 or #4 or #5)) and #6 | |
| --- | --- |
| #1  Policy/intervention | (exp Policy Making/ or exp Health Care Policy/ or exp Government Policy Making/ or exp Legislative Processes/ or Laws/) or  (policy or policies or legislation or legislative or law or legal).ti,ab. |
| #2  Obesity | (Obesity/ or Overweight/ or Body Weight/ or Body Mass Index/ ) or  (obesity or obese or overweight or bodyweight or weight or BMI or body mass index).ti,ab. |
| #3  Nutrition | (exp Nutrition/ or Food Intake/ or Food/ or Food Preferences/ or calories/ or exp Eating Behavior) or  (nutrition or nutritional or diet or dietary or food or foods or calorie or calories or calorie-dense or caloric or energy-dense or consumption or eating).ti,ab. |
| #4  Foods/food components | (exp "Beverages (Nonalcoholic)"/) or  (vegetable or vegetables or fruit or fruits or sugar or sugary or sugar-sweetened or fat or fast food or junk food or soda or drink or drinks or beverage or beverages).ti,ab. |
| #5  Infrastructure and settings | (labeling/ or exp Taxation/ or exp "Welfare Services (Government)"/) or  (menu or menus or label or labels or labelling or labeling or restaurant or restaurants or grocery or groceries or store or stores or bodega or supermarket or market or markets or greenmarkets or greenmarkets or tax or taxation or taxes or subsidy or subsidies or subsidization or subsidization or zoning or density or procurement).ti,ab. |
| #6 Limitations | limit 17 to (("0400 empirical study" or "0450 longitudinal study" or "0453 retrospective study" or "0830 systematic review" or 1200 meta analysis or 1800 quantitative study) and ("0100 journal" or "0110 peer-reviewed journal" or "0130 peer-reviewed status unknown") and (dissertation or journal article) and human and yr="2004 - 2015") |

1. **Campbell Library database (n=30)**

All text search, publication year 2004-2015, coordination groups ‘nutrition’, ‘social welfare’, and ‘Knowledge Translation and Implementation’: obesity AND policy (n=6) and nutrition AND policy (n=24)

1. **Cochrane Library databases of Abstracts of Reviews of Effects (DARE), of Systematic Reviews (CDSR), and Health Technology Assessment (HTA) (n=20)**

Search in: Cochrane Reviews (Reviews only), Other Reviews, Technology Assessments and Economic Evaluations

#1 AND #2

#1: policy:ti,ab,kw or regulation:ti,ab,kw or law:ti,ab,kw or legislation:ti,ab,kw Publication Year from 2004 to 2015, in Other Reviews and Technology Assessments (Word variations have been searched)

#2: obesity:ti,ab,kw or overweight:ti,ab,kw or nutrition:ti,ab,kw or calorie:ti,ab,kw (Word variations have been searched)

1. **DoPHER (n=149)**

Combined Freetext (All but Authors) search for (A AND B) NOT C

A: "policy" OR "policies" OR "regulation" OR "regulations" OR "regulatory" OR “intervention” OR “interventions” OR “law” OR “laws” OR “legislation” OR “legislative” OR “legal”

B: "obesity" OR "obese" OR "overweight" OR "weight" OR "bodyweight"

C: "activity" OR "exercise" OR "RCT" OR "randomized" OR "randomised"

1. **Google Scholar (n= 100)**

First 20 pages of results for the following searches:

a) With all of the words: tax; with at least one of the words: “food junk”, food , beverages, “sugar-sweetened beverage”, soda, fat, sugar; anywhere in the article; 2004-2015

b) With all of the words: healthy; with at least one of the words: store, bodega, retail, cart, vendor anywhere in the article; 2004-2015

c) With all of the words: labling; tax; with at least one of the words: calorie, menu, interpretative, "front of pack" anywhere in the article; 2004-2015

d) With all of the words: fruit, vegetable; with at least one of the words: voucher, subsidy, discount, incentive anywhere in the article; without the words: WIC; 2004-2015

e) With all of the words: healthy; with at least one of the words: procurement,"food standards"; 2004-2015

1. **Grey Literature Report in Public Health** (n=288)

Full-text search, publication year 2004-2015: obesity AND policy

1. **MedNar (n= 1,311** non-patent top results from 166,096 found in all sources)

Full Record: (((obesity OR overweight) AND ((policy OR policies OR legislation OR law OR laws OR legislation OR regulation OR regulations OR regulatory) AND (effectiveness OR impact OR effect OR effects OR evaluation))) NOT (exercise OR activity OR RCT OR clinical OR randomized OR randomised))

1. **NICE Evidence Search (n=390)**

Full text search: obesity and policy and nutrition and effectiveness; types of information: evidence summaries, policy and service development

1. **OpenGrey.eu (n=91)**

2004-2015 (policy OR policies OR law OR legislation OR legislative) AND (obesity OR overweight Or nutrition OR calorie OR calories OR food OR drink OR drinks OR food OR foods)

1. **WHOLIS (n= 427)**

Advanced search, word or phrase (obesity Or overweight Or nutrition Or weight Or bodyweight), 2004-2015

1. **US National Technical Information Service Public NTRL database (n=0)**

The website hosting this database continued to be unreachable during the research period and was therefore not included despite the original plan specified in the review protocol.
