# Supplementary material for: Improving food environments and tackling obesity: A realist systematic review of the policy success of regulatory interventions targeting population nutrition
Source: PLoS One. 2017 Aug 4;12(8):e0182581. doi: 10.1371/journal.pone.0182581 (PMC5544242; doi:10.1371/journal.pone.0182581)
Supplement: S2 File — (DOCX) [file pone.0182581.s002.docx]

| **Reference** | **Setting & time post- implementnation** | | | | **Legal basis (at time of evaluation)** | | **Study Design & Quality** | **Study Subjects** | **Prinary outcome** | | **Secondary outcomes(s)** | **Results** |
| --- | --- | --- | --- | --- | --- | --- | --- | --- | --- | --- | --- | --- |
| **Menu labeling** | | | | | | | | | | | | |
| Auchincloss et al. 2013 | | Philadelphia, PA, USA  Comparison: locations in Delaware, Maryland, and New Jersey, USA  1.5 years | | | City Council Bill 080167 2008 (Philadelphia Code, §6-102 and §6-308) | | One-time cross-sectional study with comparison group.  Quality rating: study judged poor quality due to small sample size in intervention group and restriction to one chain. | 648 adult customers at two outlets in Philadelphia and five outlets in control settings, all outlets belonging to the same chain | Calories per purchase collected from receipts. | | Saturated fat, carbohydrates, and sodium per purchase collected from receipts. | Average caloric value per purchase declined by 151 calories for food and by 155 calories for food plus beverage compared to non-regulated jurisdictions. For customers reporting having seen labels, per purchase values dropped by 400 calories, 370 mg sodium, 10 g saturated fat.  76% of customers reported noticing menu labels, of those 98% reported seeing calories, 70% reported seeing nutrient information, 50% reported seeing sodium information.  26% of all customers and 34% of those reporting having seen labels also reported using the information for purchasing decisions. |
| Bollinger et al. 2011 | | | New York City, NY, USA  Comparison: Boston, MA, USA and Philadelphia, PA, USA  Up to 10 months | | New York City Board of Health Notice of Adoption of a Resolution to Repeal and Reenact § 81.50 of the New York City Health Code (New York City Health Code § 81.50) | | Repeat cross-sectional study with control group based on sales data.  Study judged poor quality due to questionable representativeness of Starbucks customers. | 222 Starbucks locations in NYC and 94 Starbucks locations in Boston and Philadelphia > 100 million transactions and cardholder data from 7,520 customers in NYC and 3,772 in Boston and Philadelphia | Caloric value per purchase | | NA | Average calories per transaction decreased by 6 % from 247 calories to 232 calories in NYC compared to control settings, difference almost exclusively attributable to food purchases.  High calorie consumers (> 250 calories per transaction) reduced the caloric content of their average purchase by 26%.  Starbucks’ aggregate sales revenue remained stable and even increased by 3% at stores located near a Dunkin Donuts. |
| Bruemmer et al. 2012 | | | King County, WA, USA  18 months | | King County Board of Health Regulation #07-01 (Code of the King County Board of Health, Chapter 5.10) | | Repeat cross-sectional.  Study judged medium quality due to not establishing a baseline prior to policy entry into force. | 37 chain restaurants | Energy content of entrees against 2005 Dietary Guidelines | | NA | An average reduction from 818 to 777 calories was observed at 18 months compared to 6 months post-implementation. |
| Chen et al. 2015 | | | King County, WA, USA  2 years | | King County Board of Health Regulation #07-01 (Code of the King County Board of Health, Chapter 5.10) | | Repeat cross-sectional study (pre-post).  Study judged medium quality due to landline survey and self-reported usage only. | 3,132 English-speaking King County adults, data from Behavioral Risk Factor Surveillance survey | Self-reported awareness and use in making purchasing decisions | | NA | At 2 years post-implementation, the proportion of fast food chain customers who reported seeing calorie information had increased from 18.6% to 59.4% and the proportion of fast food chain customers who reported seeing and using calorie information increased from 8.1% to 24.8%.  White, higher income, and obese respondents had greater odds of seeing calorie information. Women, higher income groups, and those eating at a fast-food versus a sit-down chain restaurant had greater odds of seeing and using information. |
| Dumanovsky et al. 2010 | | | New York City, NY, USA  3 months | | New York City Board of Health Notice of Adoption of a Resolution to Repeal and Reenact § 81.50 of the New York City Health Code (New York City Health Code § 81.50) | | Repeat cross-sectional study (pre-post).  Study judged medium quality due to using self-reported behavior only for usage and not reporting clearly on refusal to participate. | 1,188 surveys pre-implementation and 1,229 surveys post-implementation at 45 outlets representing 15 different chains | Self-reported awareness and use in making purchasing decisions | | NA | At 3 months post-implementation, the proportion of fast food chain customers who reported seeing calorie information had increased from 25% of to 64%. The percentage of all customers who reported seeing and using calorie information increased from 10% to 20%. Men were more likely than women to report usage. |
| Dumanovsky et al. 2011 | | | New York City, NY, USA  9 months | | New York City Board of Health Notice of Adoption of a Resolution to Repeal and Reenact § 81.50 of the New York City Health Code (New York City Health Code § 81.50) | | Repeat cross-sectional study (pre-post).  Study judged medium quality due to sex, age, and zip code only collected post-implementation and relatively low response rate of 60% not further explored. | 15,798 transactions verified by receipt at 168 locations representing 11 chains | Calories per purchase | | Self-reported calorie use in making purchasing decisions | No statistically significant reduction in average caloric content of purchases post-implementation. There was variation between chains, with 3 chains showing statistically significant reductions (ranging from -80 to -44 calories per purchase) and one chain showing a statistically significant increase (+ 133 calories per purchase)  15% of all customers reported using the calorie information post-implementation, this sub-population reduced purchases by an average of 106 calories to 757 calories from 863 calories.  Women and customers in affluent neighborhoods were more likely to report using calorie information post-implementation. |
| Elbel 2011 | | | New York City, NY, USA  Comparison: Newark, NJ, USA  4 weeks | | New York City Board of Health Notice of Adoption of a Resolution to Repeal and Reenact § 81.50 of the New York City Health Code (New York City Health Code § 81.50) | | Repeat cross-sectional study with control group; purchases verified based on receipt and visual control  Study judged medium quality due to lack of data on participation rate. | 1,147 adults at 19 outlets of 4 chains, outlets matched on multiple characteristics. | Share of customers correctly estimating (1) recommended daily calorie intake and (2) the caloric value of their purchase | | NA | No statistically significant differential change in correct estimates of recommended daily post-implementation reported.  The share of respondents correctly estimating the caloric value of their purchase rose from 15% to 24% in New York City while declining from baseline in Newark, resulting in a statistically significant differential change post-implementation. |
| Elbel et al. 2009 | | | New York City, NY, USA;  Comparison: Newark, NJ, USA  4 weeks | | New York City Board of Health Notice of Adoption of a Resolution to Repeal and Reenact § 81.50 of the New York City Health Code (New York City Health Code § 81.50) | | Cross-sectional study with control group based on receipt and survey data.  Study judged medium quality due to lack of data on response rate. Participation rate, limited reporting of complete statistics. | 1,156 adults in low-income, minority communities, 19 outlets of 4 chains, outlets matched on multiple characteristics | Average calories per purchase | | Self-reported noticing and using of information | No differential change in average calories per purchase was observed.  Post-implementation, the share of respondents who reported seeing the information rose from approx. 15% to 54 percent in New York City, but increased only slightly in Newark (increase from approx. 10% to 18%). The total share of consumers reporting using the information in purchasing decisions also showed a differential change, rising to approx. 15% in NYC. |
| Elbel et al. 2011 | | | New York City, NY, USA;  Comparison: Newark, NJ, USA  4 weeks | | New York City Board of Health Notice of Adoption of a Resolution to Repeal and Reenact § 81.50 of the New York City Health Code (New York City Health Code § 81.50) | | Cross-sectional with comparison group study based on receipt and survey data  Medium quality due to small sample size and lack of information on response rate. | 349 children and adolescents aged 1-17 years, 19 outlets of 4 chains, outlets matched on multiple characteristics | Average calories per purchase | | Self-reported noticing and using of information | No differential change in average calories per purchase was observed.  Post-implementation, the share of respondents who reported seeing the information rose from a baseline of 0 in both regulated and control location to 57% in NYC and 18% in Newark. 9% of NYC respondents reported using the information post-implementation (no figure given for Newark despite increase in report awareness there). |
| Elbel et al. 2013 | | | Philadelphia, PA, USA  Comparison: Baltimore, MD, USA  Four months | | City Council Bill 080167 2008 (Philadelphia Code, §6-102 and §6-308) | | Repeat cross-sectional with control group, purchases verified by receipt.  Telephone survey via random-digit dialing.  Study quality judged high. | 2,083 at 23 outlets belonging to two chains  Observations  2,810 participants in a telephone survey via random-digit dialing, among those eligible (visit to a fast food restaurant in the past 3 months) the response rate was 11% | Calories per purchase | | Self-reported noticing and using of information  Self-reported visits to fast food restaurants | Post-implementation, 38% of Philadelphia consumers reported noticing calorie information, an increase of 33 percentage points relative to pre-implementation and the control locations at both time points. White respondents and those with at least some college had higher odds of seeing the information. 10% of all customers reported using labels in purchasing decision post-implementation.  There was no statistically significant differential change in calories purchased nor in the number of fast food chain visits in the restaurant survey or the telephone survey. Statistically non-significant trends in males, younger adults, blacks, and adults without a high school degree suggest increased frequentation of fast food chains post-implementation. |
| Finkelstein et al. 2011 | | | King County, WA, USA  Comparison: locations in other jurisdictions in WA, USA  Up to 7 months | | King County Board of Health Regulation #07-01 (Code of the King County Board of Health, Chapter 5.10) | | Repeat cross-sectional study with control group, calorie purchases based on sales data.  Study quality judged medium due to limitation to drive-through of one chain. | 14 outlets of one chain | Average calories per transaction. | | Changes in frequency of visits to fast food restaurants | No differential change in calories per transaction was found. |
| Krieger et al. 2013 | | | King County, WA, USA  18 months | | King County Board of Health Regulation #07-01 (Code of the King County Board of Health, Chapter 5.10) | | Repeat cross-sectional study (pre-post), purchase data based on receipts.  Study quality judged good. | 7,325 customers aged 14 and over at 50 outlets of 10 chains | Calorie intake per transaction | | Self-reported noticing and using of information | Changes in average calorie intake per transaction did not change at 4-6 months post-implementation, but showed a downward trend close to statistical significance at 18 months post-implementation (38 kcal, 95% CI (76.9, 0.8); P. 0.06 ) Changes differed between chains (decreases in taco and coffee chains, but not in burger and sandwich chains). Calories purchased in food chains declined more among women, younger customers, and affluent areas and in coffee chains for women and whites.  Self-reported noticing of labeling increased from 18.8% to 61.7% in food chains and from 4.4% to 30.0% in coffee chains. |
| Namba et al. 2013 | | | US, locations not specified with exception of New York City.  Up to 3 years | | New York City Board of Health Notice of Adoption of a Resolution to Repeal and Reenact § 81.50 of the New York City Health Code (New York City Health Code § 81.50) | | Study design designated case control.  Study quality judged medium due to lack of information about location of operation and time under regulation for intervention chains. | 9 fast-food chains | Share of healthier food options on menu (defined as less than or equal to 25% (entrees) and 10% (mains) of the Dietary Reference at study time. | | Overall caloric value of entrees and sides. | There was a statistically significant differential increase in the percentage of healthier adult entrées at restaurants in jurisdictions with menu-labeling laws. No statistically significant differential changes were observed in average nutritional values of entrees and sides. |
| Rendell and Swencionis 2014 | | | New Rochelle, NY, USA  Comparison: Stamford, CT, USA  Time post-implementation not specified. | | Local Law 13-2008 (Chapter 708 of the Laws of Westchester County) | | Study quality judged poor due to non-reporting of response rate, self-reporting of orders, small sample size, income differential between matched cities, and limitation to one chain | 245 adults at Cosi chain | Caloric value of self-reported orders. | | NA | No difference in calories per purchase. |
| Saelens et al. 2012 | | | King County, WA, USA  Comparison: and Multnomah County, WA, USA  Up to 1.5 years post enforcement | | King County Board of Health Regulation #07-01 (Code of the King County Board of Health, Chapter 5.10) | | Repeat cross-sectional study with comparison group  Study quality judged medium due to control and intervention sites sampled at different time points. | 96 outlets of different chains surveyed at all time points. | Change in healthy food environment index (NEMS-R) | | NA | There was no differential change in the availability of healthful options or facilitators of healthy eating with the exception of the mandated calorie labeling. Barriers to healthful eating decreased slightly in the regulated post-implementation compared to the control jurisdiction. |
| Taksler and Elbel 2014 | | | Philadelphia, PA, USA  Comparison: Baltimore, MD, USA  Four months. | | City Council Bill 080167 2008 (Philadelphia Code, §6-102 and §6-308) | | Cross-sectional study with control group, purchase data verified by receipt.  Study judged medium quality due to non-reporting of response rate. | 1,835 adults at 23 outlets belonging to two chains | Accuracy of estimation of calories purchased. | |  | Participants in both control and intervention location underestimated purchased calories by an average of 216-409 calories both pre- and post-implementation, with no statistically significant differential changes observed overall.  Statistically significant differential improvements were observed only in customers with at least some college education and those ordering small meals. |
| Tandon et al. 2011 | | | King County, WA, USA  Comparison: San Diego County, CA, USA  2 months | | King County Board of Health Regulation #07-01 (Code of the King County Board of Health, Chapter 5.10) | | Prospective cohort study with comparison group based on receipt and survey data.  Study quality judged medium due to small sample size and lack of clarity as to which restaurants were visited. | 133 children aged 6-11 years. | Average calories purchased for child and parent | | Self-reported awareness | The share of parents reporting seeing calorie information increased from 44% to 87% post-implementation. There was no statistically significant change in the control location.  Average calories per transaction declined by approximately 100 calories for parents, but not for children in both regulated and control location; therefore there was no statistically significant differential change between the two locations. |
| Vadiveloo et al. 2011 | | | New York City, NY, USA  Comparison: Newark, NJ, USA  4 weeks | | New York City Board of Health Notice of Adoption of a Resolution to Repeal and Reenact § 81.50 of the New York City Health Code (New York City Health Code § 81.50) | | Repeat cross-sectional with comparison group based on purchases verified by receipt data and survey data  Study judged medium quality due to significant difference in make-up of control and intervention sample and non-reporting of participation rates. | 1,170 adults at 19 outlets of four chains, intervention and control outlets matched. | Type of purchase. | | Self-reported noticing and using of calorie information; self-reported frequency of restaurant visits. | Changes in type of purchase varied: post-implementation, the share of New York consumers who ordered caloric beverages increased by 6 percentage points to 18% while consumption stayed stable in Newark at 37%. The share of New York consumers who ordered salads rose by 5 percentage points while consumption stayed stable in Newark at 3%. However, the differential change was not statistically significant for either measure.  The reported number of fast food restaurant visits per week showed a downward, but not statistically significant change post-implementation in NYC compared to Newark (where average visits also decreased).  In NYC, post-implementation, 55.5% of customers reported seeing calorie information and 14.5% reported using it in making purchasing decisions. Both groups reported statistically significant fewer average fast foods restaurant visits. |
| Wellard et al. 2015  11 months | | | New South Wales, Australia/five Australian states  Up to 9 months post mandatory compliance (previous phase-in period) | | Food Amendment Act 2010 No 108 and Food Regulation 2010: Part 2B Requirements for display of nutritional information (Part 8, Division 4 of the Food Act 2003 (NSW) and Part 2B of the Food Regulation 2010 (NSW)) | | Repeat cross-sectional.  Study rated poor for failing to provide differentiated analysis between intevention and non-interventions jurisdictions | 197 outlets belonging to the five largest fast food chains | Types and number of items with nutritional information available per outlet. | | NA | Total nutritional information (calorie counts and/or nutrient values) increased significantly nationwide post-implementation in NSW, from 178 items to 437 objects. Similarly, the proportion of stores that had no nutrition information available dropped from 32%, to 1%.  The number of items giving concrete nutrient information decreased from, 172 objects to 122 objects. |
| **Subsidies for healthy food purchases** | | | | | | | | | | | | |
| Baronberg et al. 2013 | | | Health Bucks program in New York City, NY, USA  3-4 years | | Farm Bill and USDA regulations; waiver from USDA Food and Nutrition Service required for program implementation | | Cross- sectional analysis with comparision group of EBT sales data post- Health Bucks introduction; pre-post study at 4 markets.  Study judged of good quality. | 24 farmers’ markets across 5 NYC boroughs | Daily sales via electronic benefits (ETB) | NA | | Market particpation in both SNAP via EBT and Health Bucks increased from 2006 to 2009.  Markets participating in Health Bucks averaged $170.79 more in daily EBT sales than nonparticipating markets, with the average increase attributable to a shift in sales after a 2008 expansion of the Health Bucks program.  Pre-post analysis of sales data showed significant increases in EBT sales in two out of four markets. |
| Breck et al. 2015 | | | Green carts program in the Bronx, New York City, NY, USA  5-6 years | | Local Law 9 and Notice of Adoption of amendments to chapter 6 (food units) of title 24 of the Rules of the City of New York | | Cross- sectional study  Study judged of medium quality due to limited sampling frame. | ~782 transactions at 4 Green Carts (70% response rate) | Transaction value per customer paying via SNAP compared to cash payments | Transaction value per customer using EBT for SNAP redemption | | Use of SNAP benefits was associated with an average of $3.86 more spent at Green Carts compared with cash payment. A similar difference was found at Green Carts that offered redemption via EBT. |
| Olsho et al. 2015 | | | Health Bucks program in New York City, NY, USA  3-5 years | | Farm Bill and USDA regulations; waiver from USDA Food and Nutrition Service required for program implementation | | Cross-sectional study based on survey data and ecological study.  Study judged of medium quality due to self-reporting consumption. | 2,287 respondents in customer survey in 2010; 1,025 respondents in random-digit-dial telephone survey in 2010; and 35,606 respondents in four Community Health Survey iterations (response rate 29% -36%) | Program exposure and shopping behavior | Trends in consumption of fruits and vegetables | | Greater Health Bucks exposure was significantly associated with more frequent farmers’ market shopping.  57% of farmers markets customers using Health Bucks and 37% of neighbourhood residents using Health Bucks agreed that they spend more SNAP benefits at farmers’ markets due to the program.  Self-reported fruit and vegetable consumption did not vary after introduction of the program compared to control neigbborhoods. |
| Payne et al. 2013 | | | Health Bucks program in New York City, NY, USA  4-5 years | | Farm Bill and USDA regulations; waiver from USDA Food and Nutrition Service required for program implementation | | Descriptive study based on key informant interviews and a self-administered written surveys.  Study quality not rated. | 192 farmer/vendor surveys (68% response rate) and 81 farmers’ market managers (94% response rate); 13 key informant interviews | Stakeholder views | Redemption rates for Health Nucks by distribution mehod | | A majority of farmers/vendors agreed that due to Health Bucks, (i) their profits rose (75%), (ii) they gained new customers (74%), (iii) they sold more fresh produce (72%), (iv) they had more repeat customers (70%), (v) customers bought more new or unfamiliar foods (57%)  Redemption rates for Health Bucks distributed by community organizations rose from 24% in 2005 to 69% in 2010. In comparison, EBT incentive redemption rates ranged from approximately 85% to just below 90% from 2008 through 2010. |
| Young et al. 2013 | | | Philly Food Bucks program in Philadelphia, PA, USA  Up to 2 years | | Farm Bill and USDA regulations; waiver from USDA Food and Nutrition Service required for program implementation | | Cross-sectional study based on customer surveys and sales data.  Study rated good quality. | 622 farmers market customers (estimated response rate 90%); voucher distribution (bonus incentive program to increase fruit and vegetable purchases) and redemption data. | Self-reported fruit and vegetable consumption patterns. | SNAP sales trends. | | Voucher users were 2.6 times more likely than non-voucher users to report increased fruit and vegetable consumption since becoming market customers.  Average SNAP sales per market increased 2.3-fold after the introduction of Philly Food Bucks  SNAP and voucher sales were highly correlated (2005–2009) and exceeded the $2 incentive model (provided for every $5 in SNAP benefits) by promoting extra purchases of fruit and vegetables amounting to $3.75 in SNAP sales in 2010 and $2.74 in 2011. |
| **Food infrastructure improvement** | | | | | | | | | | | | |
| Fuchs et al. 2014 | Green Cart program in New York City, NY, USA  approx. 5 years | | | Local Law 9 and Notice of Adoption of amendments to chapter 6 (food units) of title 24 of the Rules of the City of New York | | Descriptive study based on the assumption of baseline 0 pre-intervention.  Methodology combined vendor census, comprehensive vendor survey, and consumer and customer survey at four Manhattan cart locations.  Study quality judged poor in framework of conventional pre-post study appraisal due to descriptive character. | | Green Carts and their vendors and customers. | Green Cart operation and placement | | NA | 142 vendors operating 166 carts identified against 492 active permits  95% of vendors located near a bus stop, 55% near a subway.  31% of vendors operate year round  27 % had EBT machines  Clusters observed around areas with high pedestrian traffic in shopping districts and near other vendors.  Most Green Carts are located in areas with relatively low produce density.  Most customers earn less than $50,000; 44% live at or below the poverty line, 18% percent reported receiving public assistance; 50% reported being “always” or “sometimes” worried about having enough money to buy fresh fruits and vegetables. <1% consider green carts their produce source, yet 63% use them at least once a week. |
| Kerker et al. 2012 | Green Cart program in New York City, NY, USA - Green Cart neighborhoods and matched comparison neighborhoods  3 years | | | Local Law 9 and Notice of Adoption of amendments to chapter 6 (food units) of title 24 of the Rules of the City of New York | | Cross-sectional study with comparison group; ecological study based on the community Health Survey (CHS).  Study quality for comparison group study rated fair due to missing information on measurement methods and lack of reporting of statistical details. | |  | Number of allocated green cart permits active. | | Proportion of establishments selling more than 10 types of fruits and vegetables; quality of produce. | 50% of available green cart permits were active (on paper)  The proportion of establishments selling more than 10 types of fruits and vegetables rose from 31% to 38% in Green Cart neighborhoods, but declined in non-Green Cart neighborhoods to a still higher level of 47%.  Produce quality declined 8% to 88% in Green Cart neighborhoods, but stayed the same in non-Green Cart neighborhoods at 94%.  There was no statistically significant increase in reported fruit and vegetable consumption on the previous day between 2008 and 2011. A statistically insignificant, slight upwards trend was reported in Green Cart neighborhoods. |
| Li et al. 2014 | Green Cart program in New York City, NY, USA  Approx. 5 years | | | Local Law 9 and Notice of Adoption of amendments to chapter 6 (food units) of title 24 of the Rules of the City of New York | | Cross-sectional study based on the assumption of baseline 0 pre-intervention; comparison of location of actual green carts with non-taken locations within eligible zones.  Study quality judged good. | | Green carts and census tracts | Predictors for green cart placement; differences in neighborhood characteristics between of Green Carts locations and candidate sites | | NA | Green Carts were more likely to be next to a subway stop (OR 59.3 (30.7–114.4)), near a large employer (OR 6.3 (3.7–10.8)), in a more population-dense area (OR 3.2 (1.7–6.0)), and near retail considered healthy (OR 2.4 (1.3–4.6))  Compared with the characteristics of candidate sites, i.e. neighborhoods designated by city regulations for green cart permits which were not taken up, 29% fewer green carts were actually located in areas designated as underserved. |
| Lucan et al. 2011 | Green Cart program in the Bronx, New York City, NY, USA  Approx. 2 years | | | Local Law 9 and Notice of Adoption of amendments to chapter 6 (food units) of title 24 of the Rules of the City of New York | | Descriptive study based on the assumption of baseline 0 pre-intervention.  Study quality judged poor in framework of conventional pre-post study appraisal due to descriptive character and explorative approach to study purpose. | | 61 Green Carts | Location of green carts | | Compliance with program requirements | Green Carts were clustered around medical, academic that were inaccessible to 43% of their area.  3 of 61 Green Carts were observed selling outside the specified area, with; 3 out of 21 Green Carts sold cookies and/or sugar-sweetened beverages. |
| Sturm and Hattori 2015 | Ban on new standalone fast-food restaurants in South Los Angeles, CA, USA  Comparison: other parts of the City of Los and Los Angeles County  4.5 years | | | Council File No. 07-1658.(2007, interim) and Council File No. 10-1843 (2010, extension) | | Cross-sectional study with comparison group; ecological approach for the examination of diet and BMI trends using data from the California Health Interview Survey  Study rated fair quality due to absence of pre-implementation measures for food permits and lack of reporting on statistical analysis of differences between jurisdictions. | | New food permits | Change in food permits | | Change in average dietary intake and BMI | About 10% of current (at study end) food outlets opened under new rule.  Food environment changes:  1. In comparison to control locations, a higher share of new permits was awarded to small food markets (+~20%) and a smaller share to large non-chain restaurants (-~20%).  2. In comparison to control locations, new licensees that were fast food and other chains made up a slightly greater share of the existing food environment and small food markets made up a slightly smaller proportion, but the changes were statistically insignificant  Diet and BMI changes by jurisdiction: no statistically significant differences after controlling for individual- and census tract-level characteristics |
| **Taxation of unhealthy foods and beverages** | | | | | | | | | | | | |
| Berardi et al. 2012 | Tax of 7.16 euros per hectoliter (or 0.076 euros per 1 liter) beverage, France  6 months | | | Loi de finances 2012 (Art. 1613 ter., Code général des impôts) | | Equivalent of repeat cross-sectional study based on price reports.  Study quality judged good. | | Prices of 850 different beverage products | Price change in relation to tax | | NA | 6 months post-implementation, soda prices fully reflected the tax and fruit drinks and flavored waters partially increased in price. |
| Biro 2015 | Taxation of various unhealthy foods and beverages, Hungary  16 months | | | 2011 public health product tax (Népegészségügyi termékadó) | | Equivalent of repeat cross-sectional study based on household panel survey  Study quality judged medium due to use of household panel (possible selection bias) and inability to differentiate between taxed and untaxed purchases. | | 44,608 household level observations | Purchased quantities of processed and unprocessed products | | NA | After introduction of the junk food tax, purchased quantities of processed foods declined by 3.4% and purchased quantities of unprocessed foods increased by 1.1% (statistically insignificant). |
| Bodker et al. 2015 | Tax based on saturated fat content, Denmark  15 months | | | Act on a Tax on Saturated Fat in Specific Food | | Equivalent of repeat cross-sectional study based on retail outlet sales data.  Study quality judged good. | | Total sales of twelve foodstuff categories from all but 2 retail chains in Denmark | Change in sales. | | Extrapolated change in nutrient consumption and disease risk | During the tax, the total sale of the 12 taxed foodstuffs included in the study decreased by 0.9%, but increased by 1.3% pre-implementation and post-abolition of the tax. There were variations between categories.  Sales changes are estimated to translate into an increase of population heart disease by 0.2%-0.5% due to a decrease of both saturated fat and unsaturated fat intake. |
| Jensen and Smed 2013 | Tax based on saturated fat content, Denmark  Nine months | | | Act on a Tax on Saturated Fat in Specific Food | | Equivalent of repeat cross-sectional study based on household.  Study quality judged medium due to use of household panel (possible selection bias). | | Data from approximately 2,000 households | Change in purchases. | | Extrapolated change in product consumption. | Sales of butter, butter blends, margarine and oils decreased by 10%-15%, partially attributed to hoarding effect prior to entry into force.  Some post-tax price increases were higher at discount stores than at supermarkets; some demand appeared to have shifted from higher end supermarkets to lower-end discount stores.  Extrapolated reduction in total consumption of the four fat products by 3.66 g per individual per day. |
| Jensen et al. 2015 | Tax based on saturated fat content, Denmark  1 year | | | Act on a Tax on Saturated Fat in Specific Food | | Study judged good quality. | | Sales data from 1,293 stores (spanning different chains) owned by Coop Denmark | Change in price and sales for three product groups: minced beef, cream, sour cream. | | Extrapolated change in consumption | Prices of minced beef and cream were higher post-implementation, but no consistent pattern was observed for sour cream prices. Price chances were stronger for medium-fat and weakest for low-fat varieties of minced beef and cream.  Sales changes were extrapolated to a decrease of 4-6 % in the intake of saturated fat from minced beef and cream, but no significant effect was found for sour cream. |
| **Government food standards** | | | | | | | | | | | | |
| Cradock et al. 2015 | Boston, MA, USA  2 years | | | | Executive Order of Mayor Thomas M. Menino: An Order Relative to Healthy Beverage Options (2011) | | Repeat cross-sectional study with comparison group  Study quality was judged fair due to lack of statistical analysis comparing differences between exposure and control group. | Boston city agencies (22 properties representing 31 access points) and local recreation sites as a comparator | Healthy beverage availability (changes in categories ‘red’ = over 12 g sugar per 12 oz; ‘yellow’ = 6 g to 12 g of sugar per 12 oz or containing artificial sweeteners; green = 0 to 5g of sugar per 12oz) | | Average calories per beverage | The share of beverage offerings classified as red declined by 27.8% (P <.001) and yellow beverages increased by 26.1% (<.001). The share of green beverages did not change to the degree of statistical significance, but showed a slight upwards trend. At the comparison sites, the share of red beverages remained constant at 83.3% and in city recreational facilities their share dropped from 61.1% to 30.4%.  Average caloric content declined by 48.6 kcal, from 88.1 kcal to 39.5 kcal (55.2% decrease, P<0.01) compared to a drop from 123.3 kcal to 83.3 kcal (-40 kcal, 32.4% decrease) in city recreational facilities and from 140.2 kcal to 122.1 kcal (-18.1 kcal, 12.9% decrease) at the state-controlled site. |
| **Product labeling** | | | | | | | | | | | | |
| Fabiansson 2006 | New South Wales, Australia | | | | Australia and New Zealand Food Standards Code | | Descriptive study of labeling accuracy.  Study quality not rated. | 350 samples of 70 different products | Accuracy of labeling against laboratory test | | NA | 7% of samples matched the nutritional information given on the label, with 70% of information for individual nutritional components across products within ± 20%. |
